# Good news–bad news: the Yin and Yang of immune privilege in the eye

**DOI:** 10.3389/fimmu.2012.00338

**Published:** 2012-11-27

**Authors:** John V. Forrester, Heping Xu

**Affiliations:** ^1^Laboratory of Immunology, Lion’s Eye Institute, University of Western AustraliaPerth, WA, Australia; ^2^Ocular Immunology Laboratory, Section of Immunology and Infection, Institute of Medical Sciences, University of AberdeenAberdeen, UK; ^3^Centre for Vision and Vascular Science, Institute of Clinical Science-A, Queen’s University BelfastBelfast, UK

**Keywords:** immune privilege, para-inflammation, eye

## Abstract

The eye and the brain are prototypical tissues manifesting immune privilege (IP) in which immune responses to foreign antigens, particularly alloantigens are suppressed, and even completely inhibited. Explanations for this phenomenon are numerous and mostly reflect our evolving understanding of the molecular and cellular processes underpinning immunological responses generally. IP is now viewed as a property of many tissues and the level of expression of IP varies not only with the tissue but with the nature of the foreign antigen and changes in the limited conditions under which privilege can operate as a mechanism of immunological tolerance. As a result, IP functions normally as a homeostatic mechanism preserving normal function in tissues, particularly those with highly specialized function and limited capacity for renewal such as the eye and brain. However, IP is relatively easily bypassed in the face of a sufficiently strong immunological response, and the privileged tissues may be at greater risk of collateral damage because its natural defenses are more easily breached than in a fully immunocompetent tissue which rapidly rejects foreign antigen and restores integrity. This two-edged sword cuts its swathe through the eye: under most circumstances, IP mechanisms such as blood–ocular barriers, intraocular immune modulators, induction of T regulatory cells, lack of lymphatics, and other properties maintain tissue integrity; however, when these are breached, various degrees of tissue damage occur from severe tissue destruction in retinal viral infections and other forms of uveoretinal inflammation, to less severe inflammatory responses in conditions such as macular degeneration. Conversely, ocular IP and tumor-related IP can combine to permit extensive tumor growth and increased risk of metastasis thus threatening the survival of the host.

## INTRODUCTION

The first Ophthalmology textbook in English, written in the first half of the nineteenth century, contained a description of sympathetic ophthalmia (SO), an intraocular inflammatory disease which develops in the fellow eye several months after penetrating injury to the first eye (Mackenzie, 1830). SO was a pre-eminent example of “horror autotoxicus” (reviewed in Mackay, 2010) and the search was on to define the autoantigen(s) (Faure, 1980), many of which are located in the retina (Wacker, 1991). At the same time as immunity and autoimmunity were being recognized, the remarkable acceptance of corneal allografts compared to skin allografts (ZIrm, 1906), which had been reported in the early part of the twentieth century, allowed Medawar to formulate the concept of immune privilege (IP). IP was a property of certain tissues (specifically the eye and the brain; Medawar, 1961) in which foreign antigens placed in those tissues failed to evoke a conventional immune response. Such tissues were seen to be afforded a level of protection from immunological damage (termed IP).

The concept of IP has since been extended and is now regarded as a relative term, not unique to the eye or brain; it is a property of many tissues, develops *de novo* in accepted vascularized grafts (Cobbold, 2009; Huang et al., 2010) and constitutes part of the immune response to tumors (Mellor and Munn, 2008). Ocular IP is inducible and transferable (through adoptive transfer of CD8^+^ T regulatory cells (Tregs) –- infectious tolerance; Griffith et al., 2011) and thus has informed immunology generally on regulatory mechanisms.

Despite ocular IP, autoimmune and immune-mediated diseases of the eye occur with demoralizing frequency; for instance, 5 year survival rates of corneal allografts in humans are lower than those of solid organ grafts (Williams and Coster, 1997), although this statistic can be somewhat misleading since most corneal allografts in humans are performed without tissue matching (see below); also, both innate and adaptive immune mechanisms underlie several blinding ocular diseases, the scourge of populations word-wide, such as age-related macular degeneration (AMD), infectious corneal blindness, glaucoma and the “Cinderella” disease, uveitis (see **Box 1**).

**Box 1. Uveitis.**

Terminology for Uveitis is confusing and as a result the condition has been somewhat neglected as a global cause of blindness (thus it is a “Cinderella” syndrome) mainly because clinicians have had difficulty reaching agreement as to what constitutes uveitis. However, a recent initiative is aimed at developing international criteria for the various entities that come under the umbrella of uveitis (Standardization of Uveitis Nomenclature, SUN; Jabs et al., 2005).

**FIGURE 1:**
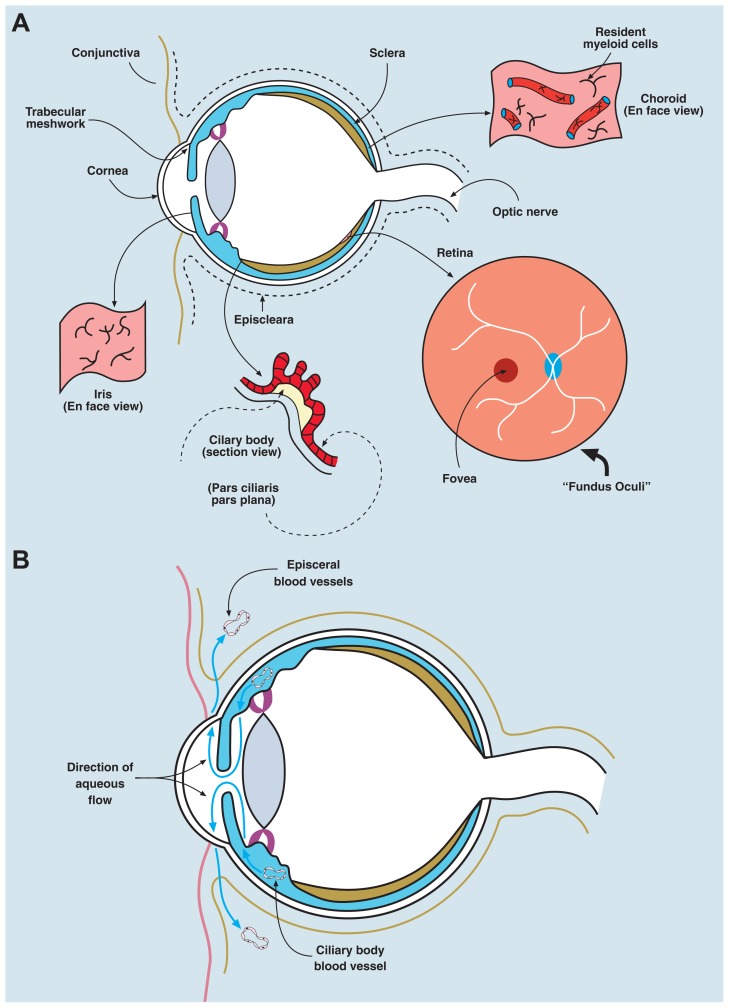
**Anatomy/physiology of the eye to include ocular immune cells**. **(A)** The eye is composed of three layers: an outer layer (cornea/sclera), an inner layer (retina), and a middle layer (uvea, a continuous structure comprising iris, ciliary body, and choroid). The anterior chamber lies between the iris and the cornea, the posterior chamber between the lens and the iris, and the vitreous cavity (containing the vitreous gel, a type II/XI collagenous avascular extracellular matrix) describes the main chamber of the eye behind the lens. The human eye maintains a pressure between 10 and 20 mm Hg, which is generated by the unidirectional flow of fluid (aqueous humor secreted by the ciliary body) from the posterior chamber into the anterior chamber, and leaving the eye via the trabecular meshwork, to drain into the episcleral veins. The “fundus oculi” is the view of the retina/choroid seen through the ophthalmoscope; the central fovea/macula is a cone photoreceptor-rich area, 500 microns in diameter, subserving central visual acuity. The remaining retina provides peripheral vision (visual field) and all visual information is transmitted through retinal neuronal cells, via the optic nerve, which synapse in the lateral geniculate body intracranially. The uvea contains a network of resident innate immune cells (DCs and macrophages) and is highly vascular (seen in section and *en face* views in the figure). The retina contains few conventional resident myeloid cells, but has a population of microglial cells (see text). Normal ocular tissues are devoid of lymphocytes. The cornea contains a population of passenger leukocytes mostly in its peripheral rim as well as some lymphatics in this region connecting with lymphatics in the conjunctiva. **(B)** Eye health is dependent on having a normal intraocular pressure, which is maintained between 12 and 20 mm Hg by the flow of aqueous fluid from the posterior chamber of the eye (the space between the posterior surface of the iris and the anterior surface of the lens) and the anterior chamber (the space between the posterior surface of the cornea and the anterior surface of the iris). The vitreous cavity is the intraocular compartment behind the lens and in front of the retina. Aqueous fluid is secreted by the epithelial cells of the ciliary body into the posterior chamber and flows through the pupil of the iris into the anterior chamber to drain through the trabecular meshwork at the angle of the eye between the iris and the cornea, into the subconjunctival space, to be finally removed by interstitial fluid flow into the episcleral veins and the subconjunctival lymphatics.

The term “Uveitis” is a misnomer since it suggests that the focus of inflammation is the uvea. Discrete parts of the uvea can be affected separately: the iris (iritis), ciliary body (cyclitis, iridocyclitis), choroid (choroiditis), or entire uvea (panuveitis; see **Figures 1A**,**B**). However, the triggering antigens (either foreign or self-antigens from retina, lens, cornea) can be located in any of the tissues, including the uveal tract itself. The most potent autoantigens have been identified in the retinal photoreceptors. Accordingly, uveitis (uveoretinitis) is also classified under the term “Intraocular Inflammation,” and sub-classified as to whether it affects the anterior segment of the eye (“anterior segment intraocular inflammation,” ASII) in which it is restricted to the cornea, anterior chamber, iris, ciliary body, and lens, or it selectively affects the posterior segment, which includes the pars plana region of the ciliary body (pars planitis), the vitreous gel (vitritis), the retina (retinitis), the retinal vessels (retinal vasculitis), the choroid (choroiditis), or the optic nerve (papillitis, optic neuritis; posterior segment intraocular inflammation, PSII).

Uveitis according to the SUN criteria is classified by its underlying cause and then according to its anatomic location (**Table 1**).

**Table 1:** Classification of uveitis (SUN criteria) with some examples.

Classification	Type of uveitis/uveoretinitis/intraocular inflammation	
	Infectious	Non-infectious
Anterior	Viruses e.g., HSV, CMV, VZV	HLA B27-associated uveitis
Intermediate	Toxocara Toxoplasmosis	Pars planitis Idiopathic vitritis Intermediate uveitis
Posterior	Toxoplasmosis Tuberculosis Syphilis Lyme disease	Retinal vasculitis Multifocal choroiditis PIC* Behcet’s disease Vogt–Koyanagi–Harada disease Panuveitis
Candida	All of above	All of above

Ocular IP has been reviewed several times recently (Caspi, 2006; Niederkorn, 2006; Ferguson and Griffith, 2007; Forrester et al., 2008b). This review therefore will focus on the place of IP as an immunoregulatory, tolerance-inducing mechanism, and discuss its limitations in the context of sight-threatening diseases.

## IMMUNOLOGICAL PROPERTIES OF THE EYE

### CELLS AND TISSUES

Several ocular tissues such as the uvea (middle layer of the eye comprising the iris, ciliary body, and choroid), the cornea, the conjunctiva, and periocular fascia (Forrester et al., 2008a; see **Figure 1**), contain rich networks of innate immune cells (bone marrow-derived resident macrophages and dendritic cells, DCs) which, together with the parenchymal cells, secrete a wide range of mediators which underpin IP (Forrester et al., 2008b, 2010). The retina contains specialized myeloid cells (microglia), similar to brain microglia, recently reported to originate from yolk sac precursors (Ginhoux et al., 2010). In addition, the central (around the optic nerve) and peripheral (at pars plana, **Figure 1**) rims of the retina contain a small population of DCIR^+^ MHC Class II^hi^ DCs, as does the corneal periphery (Xu et al., 2007b).

Recently, a small population of retinal DCs has been described in mice expressing CD11c-GFP (Heuss et al., 2012) although the specificity of CD11c for DCs is open to question. The central cornea has few DCs but contains MHC Class II^+^ macrophages (Brissette-Storkus et al., 2002; Sosnova et al., 2005; Kuffová et al., 2008) while peripheral corneal epithelial Langerhans cells and stromal Langerin^+^ cells also reside in the cornea (Hattori et al., 2012). The lens contains no myeloid cells while the normal extravascular tissue of the eye is devoid T or B cells.

### BLOOD–OCULAR BARRIERS

The intraocular compartments (**Figure 1B**) are separated from the blood and lymphatic circulations by the blood–aqueous barrier and the blood–retinal barrier (BRB; Forrester et al., 2008a).

#### Blood–Aqueous Barrier

Blood–aqueous barrier has two components – tight junctions between the endothelial cells of the ciliary blood vessels and similar junctions between the lining epithelial cells (**Figure 1**). The epithelial cells of the ciliary body maintain the intraocular pressure by pumping fluid which drains through the porous trabecular meshwork of the anterior chamber into the blood and lymphatic vessels of the episclera (**Figure 1B**). The sclera itself, like the central cornea, is avascular.

#### Blood–Retinal Barrier

**FIGURE 2:**
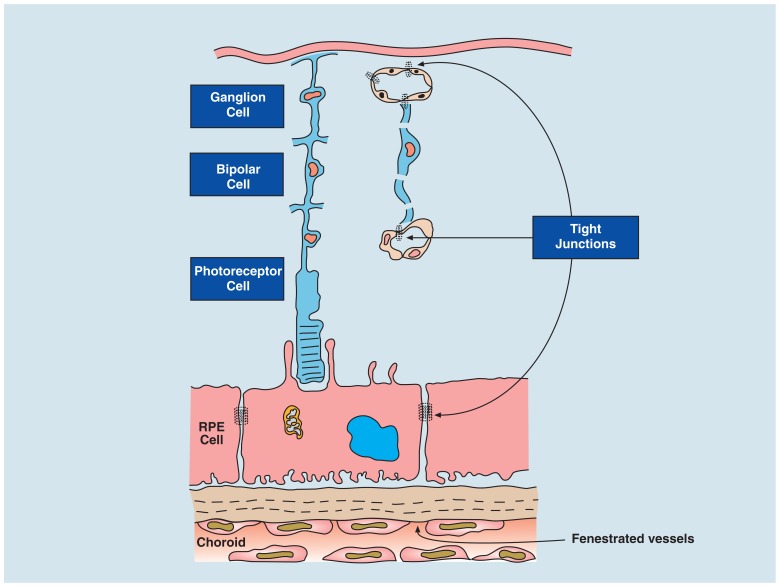
** Blood–retinal barrier (BRB)**. The BRB is created by tight junctions at two sites: between endothelial cells of the retinal vessels that supply the inner retina (ganglion cells and bipolar cells) and the retinal pigment epithelium (RPE cell; which filters blood from the fenestrated, leaky choroidal vessels). The RPE regulates two-way transport of fluid, nutrients, and waste between the outer retina (photoreceptors) and the fast flowing, high volume choroidal bloodstream. The choroid stroma contains resident innate immune cells to maintain homeostasis in the outer retina (see Forrester et al., 2010) as well as fibroblasts and melanocytes. Breakdown of the BRB can thus occur either at the retina vessels or at the RPE layer.

Blood–retinal barrier also comprises two components –- tight junctions of the retinal vessels and of the retinal pigment epithelium (RPE; **Figure 2**). The RPE is a terminally differentiated layer of neuroectoderm-derived cells formed embryologically by cells of the developing outer layer of the optic cup (Kim and Kim, 2012) whose function is to maintain the physiology of the photoreceptors and remove waste products (see below).

### OCULAR CONNECTIONS TO SECONDARY LYMPHOID TISSUES

**FIGURE 3:**
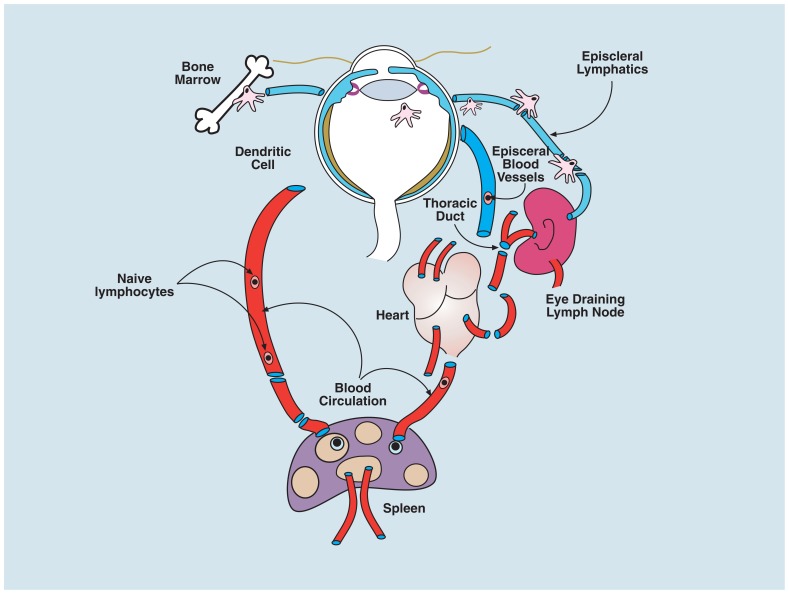
** Circulation of immune cells to and from the eye**. Myeloid cells (macrophages and DCs) traffic from the bone marrow to the eye via the blood and populate some ocular tissues, predominantly the uvea (iris, ciliary body, and choroid); a few cells enter the peripheral retina and cornea. When ocular tissues are perturbed, bone marrow-derived cells, carrying antigen from the eye, can be found in the eye-draining lymph node (DLN; see text). However whether there is transport of steady-state antigen to the DLN is not known. Both cell-associated and soluble antigen injected into the eye, or applied to the abraded cornea, can be detected in the spleen after several hours. Resting T and B cells circulate normally through the uveal blood vessels of eye but do not cross the blood–ocular barriers; it is presumed they communicate with eye-derived antigen presenting cells in the secondary lymphoid tissues (spleen and lymph node) and respond appropriately to promote tolerance or immunity as in other tissues (see text).

Although the periocular tissues such as the conjunctiva and the episclera (see **Figure 1**) contain lymphatics, the intraocular compartment of the eye lacks traditional lymphatics. Aqueous fluid from the anterior chamber, presumably containing soluble antigen shed physiologically, drains *via* episcleral blood vessels (aqueous veins) through the venous circulation to thymus, liver, and spleen (**Figure 3**). There is also a site-specific eye-draining lymph node (DLN) which receives soluble and cell-associated antigenic material from the eye (Plskova et al., 2002; Kuffová et al., 2008). Fluid also tracks by transscleral flow from the vitreous cavity across the retina driven by a RPE Na^+^K^+^ ATPase pump which, when damaged, causes subretinal fluid accumulation (Forrester et al., 1990), but the likelihood of shed ocular antigens reaching lymphatics through this route is low, given the extremely high flow rates of blood through the choroidal vessels (Forrester et al., 2008a).

### NEURAL CONNECTIONS

Aside from the neural connections to the brain *via* the visual pathways, for which there are several types of photoreceptors, as well as light-sensitive melanopsin-containing neurons generating circadian rhythms (Do and Yau, 2010), the eye has a full complement of motor, sensory, and autonomic nerves. Some provide, or respond to, immunoregulatory moieties with IP-promoting properties, including the neuropeptides, αMSH, CGRP and VIP, PACAP, melanocortin and retinoids (reviewed in Forrester et al., 2008b). Others provide routes for immune attack and evasion as in herpes virus infection of the cornea (*via* the Vth cranial nerve). CD8 T cells have a unique role in maintaining HSV latency at the trigeminal ganglion (Sheridan et al., 2007; Frank et al., 2012).

## THE HAZARDOUS NATURE OF OCULAR IMMUNE PRIVILEGE

The term IP was coined to indicate that certain tissues or cells have an advantage over others, allowing them to modulate immune responses to foreign antigens or to be regarded themselves as non-immunogenic when transplanted (e.g., stem cells; Robertson et al., 2007; Zhou and Caspi, 2010). However, IP as such carries risks to the host since it permits foreign antigens/organisms to “hide” in IP sites (Wekerle, 2006).

### WHAT IS IMMUNE PRIVILEGE?

Different levels of IP apply to many tissues, including some which are normally expected to mount full blown immune responses to foreign antigens (skin, lung, and gut). Conceptual shifts in the self/non-self paradigm underpinning adaptive immunity have emerged from evidence that innate immune recognition of foreign antigens has some degree of specificity [*via* pathogen-associated molecular patterns (PAMPs) and pathogen recognition receptors (PRRs; Kumar et al., 2011)]. This idea has evolved beyond selective recognition of pathogens, to recognition of “Danger” by the host (Matzinger, 2001; Miller et al., 2011), and to the notion that different tissues contribute variably to immune regulation particularly of the effector response, depending on which specific structures or functions are threatened (Matzinger, 2007; Matzinger and Kamala, 2011). While the Danger hypothesis has greatly broadened our understanding of how immunological responses are initiated, a certain caution might be exercised since it is difficult to define “Danger” in molecular or cellular terms. Accordingly, since IP itself as a concept is contingent upon the self/non-self paradigm of adaptive immunity, it is timely to review exactly what IP means.

At its core, IP is a form of tolerance expressed through tissue-specific properties (see above), is shown to be inducible toward foreign and alloantigens, and is assumed to underpin tolerance to ocular self-antigens. As a general hypothesis therefore, we can consider tissue-centered immunological tolerance as additional to the well-established central and peripheral tolerance mechanisms (Matzinger, 2007). Tissue-centered tolerance (TCT; Matzinger and Kamala, 2011) varies with the nature, properties, and vascularity of the tissue and is optimal in tissues such as eye and brain. In other tissues, TCT or IP can be induced, for instance as in accepted allografts and even in the stem cell niche (Robertson et al., 2007; Cobbold, 2009; Waldmann, 2010; Fujisaki et al., 2011). Interestingly, in these environments Treg induction seems to be the main mechanism of immune tolerance. As such, tissues which depend on their intrinsic properties for immunological protection or privilege (IP) are at greatest risk when tolerance is breached.

Several mechanisms are proposed to explain ocular IP including sequestration of antigen from the developing immune system behind blood–ocular barriers (immunological ignorance), lack of lymphatics, absence of MHC Class II^+^ professional antigen presenting cells (APCs; Hamrah and Dana, 2007; Forrester et al., 2010; negates CD4 T cells), lack of expression of MHC Class I on tissue cells (negates CD8 T cells), expression of Qa-1 and HLA-G/E (negates NK cells), presence of immune modulators such as TGFβ, CD200, CD55, and CD46, decay-acceleration factor (DAF; modifies APCs), and lack of thymic expression of tissue antigens (no autoreactive T cells to escape to the periphery; Forrester et al., 2008b; Zhou and Caspi, 2010).

In addition, parenchymal cells of the eye have the capacity, at least *in vitro*, to exert T cell suppressive activity (Caspi et al., 1987; Ferguson and Griffith, 2007) as well as generate Tregs (Taylor et al., 1997; Yoshida et al., 2000; Zamiri et al., 2007; Sugita et al., 2010). Expression of molecules such as FasL (Ferguson and Griffith, 2007), PDL1 (Usui et al., 2008), CTLA4 (Sugita et al., 2006), and the recently described CTLA2 (Sugita et al., 2008) by ocular cells particularly the iris, ciliary body, and RPE cells generate an immunosuppressive microenvironment (Stein-Streilein, 2008). The PD-1 pathway is interesting since it appears to have the capacity to promote T effector cell death similar to the Fas/FasL pathway, but also has the potential to promote Tregs (Francisco et al., 2010). Recently, retinoids (specifically retinoic acid, RA) have been implicated in the induction of Tregs by the RPE *in vitro* (Kawazoe et al., 2012). RA produced by CD103^+^ lamina propria DCs has been identified as a major inducer of intestinal Tregs and this property of DCs has been extended to non-intestinal DCs (for review, see Nagy et al., 2012). In these conditions, the Treg-inducing effects of RA required TGFβ (Cong et al., 2009). Recent data showed that vitamin A-deficient mice were unable to convert naїve T cells to Tregs in the uninflamed eye *in vivo* which was interpreted as being due to an absence of locally produced RA, presumably by intraocular DCs (Zhou et al., 2012). However, in conditions of active experimental autoimmune uveoretinitis (EAU), committed T effector cells could not be converted to Tregs suggesting that this component of IP was lost during inflammation. Previous studies had, in fact, shown that RA promoted the immunogenicity of DCs in a pro-inflammatory environment (Geissmann et al., 2003) and this may explain the results from the above experiment in uveitis.

A potential role of RA in IP in the eye is a logical conceptual extension given the role and high concentrations of retinoids in the visual cycle. The RPE specifically expresses one of the enzymes (RALDh10) required for metabolizing retinal (vitamin A) to RA, and all-trans retinal itself has recently been shown to induce Tregs *in vitro* (Jeon et al., 2012). However, most of the retinoids in the RPE, if not used in the visual cycle, are stored as inactive retinyl esters, and are converted to retinal by RALDh10 if vitamin A blood levels decline. *In vivo* evidence that visual cycle retinoids themselves participate in IP is lacking, probably because their escape from the visual cycle is tightly regulated. Indeed it has been shown that accumulation of all-trans-retinal in mice lacking the ABCA4 transporter (ATP-binding cassette transporter 4) and RDH8 (retinol dehydrogenase 8) are liable to retinal degeneration, particularly that induced by light (Maeda et al., 2009). However, there are large networks of ocular tissue-resident DC which are likely to be a source of retinoids for Treg induction (Forrester et al., 2010). Indeed this may be a universal property of all tissues related to tissue-resident, homeostasis-promoting, self-tolerizing DCs, which is dependent on the density and phenotype of the DC, and is at a high level in the eye. Interestingly, Tregs are important for maintaining IP in the brain and they maintain this function even in the face of acute viral encephalitis, thus minimizing bystander damage and presumably preventing autoimmunity associated with the viral infection (Cervantes-Barragan et al., 2012). Induction of Tregs by DC in allografts and other sites has been described as a form of acquired IP and has been attributed in part to the expression of indoleamine oxidase (IDO; for review, see Huang et al., 2010; Kushwah and Hu, 2011) probably underscoring the fact that there are multiple potential routes for induction of Tregs, but more importantly highlighting the link between IP and Treg induction. Interestingly, RA- and TGFβ-mediated Treg induction does not carry over to IL-10 Tr1 regulatory cell induction, in which these molecules do not have any effect and may in fact have a negative role (Maynard et al., 2009).

How Tregs might mediate privilege is unclear. Mice with defects in central tolerance (such as those deficient in the autoimmune regulator gene *Aire* required for negative selection of certain tissue-specific antigens presented by mTECs) develop mild to moderate ocular inflammation as part of a multiple autoimmune diathesis (Anderson et al., 2002; DeVoss et al., 2006). However, *Aire* is also involved in induction of natural Tregs in the thymus (Aschenbrenner et al., 2007) and *Aire* has also been detected in the periphery, both factors indicating that this gene probably does not have a restricted role in central tolerance (Metzger and Anderson, 2011). Mice with defined immunodeficiencies involving Tregs are not known to have ocular pathology which would suggest that either Tregs do not contribute to ocular IP or that tissue-centered “IP-like” mechanisms (TCT) are sufficient to sustain tolerance in the eye. This has however, to our knowledge, not been directly tested in Treg-deficient mice, using conventional models of ocular autoimmunity, such as EAU. Despite this, patients with uveitis are reported to have decreased levels of circulating Tregs which suggests that peripheral tolerance, if not IP, is necessary to maintain immunological homeostasis. Tregs occur as part of the infiltrating inflammatory cell population in the retina in EAU, indicating that they almost certainly play a role. Since TCT is one aspect of tolerance generally, it may be somewhat semantic to attribute exclusivity in these mechanisms.

It can be seen that there are many possible mechanisms which explain the immunosuppressive properties of the eye associated with IP, all of which have been shown to be only partially validated, if functional at all *in vivo*; moreover, one single mechanism is unlikely to account fully for ocular IP; instead, each probably contributes to overall immune homeostasis. In this context, the eye’s IP properties offer strong supportive evidence for Matzinger’s TCT hypothesis but as discussed below, in many circumstances IP/TCT is insufficient to fully protect the eye from Danger.

### DOES IP HAVE MEMORY?

Adaptive immune responses to foreign antigens are characterized by memory. Tolerance to self-antigen involves antigen-specific responses leading to deletion, peripheral anergy, and/or regulation with memory. Tolerance operates when there is risk of self-antigen exposure to the immune system, for instance during apoptotic turnover of cells, by processes which suppress immune-mediated inflammation and encourage “silent” clearance of cellular debris. Much of this activity is conducted in secondary lymphoid tissues like the liver and spleen, which drain the interstitial fluids *via* lymphatic and blood circulations.

Tolerance to nominal antigens is demonstrable when, after exposing the organism to antigen, an immune response to that antigen cannot be induced on re-challenge. In some circumstances, an immune response can be elicited on re-challenge but is attenuated or modified: the latter circumstance is known as immune deviation. Tolerance is an active process involving T cell proliferation followed by apoptosis (activation-induced cell death, AICD) and clearance of cell debris by resident tissue scavenger cells. However, if the initial encounter with antigen involves cell necrosis and/or “adjuvant” effects of microorganisms *via* PRR activation and induction of IL-1/IL-18 *via* the inflammasome or other mechanisms (van de Veerdonk et al., 2011), an active pathogenic immune response with concomitant inflammation and bystander tissue damage may occur.

There are several routes of antigen (Ag) administration for inducing tolerance: intravenous (iv), subcutaneous (s/c), mucosal (oral/nasal/conjunctival), intraperitoneal (ip), and intraocular (io). Io injection involves antigen uptake by APC (Dullforce et al., 2004) and induction of antigen-specific T cells, followed by T cell and APC apoptosis *via* Fas/FasL (Green and Ferguson, 2001). Systemic tolerance to that antigen can then be demonstrated by antigen re-challenge in a delayed-type hypersensitivity (DTH) skin test (as originally shown for alloantigens by Medawar, 1948). This response is antigen-specific and thus has “memory,” requires a minimum period of 3 days to develop, and is dependent on an intact oculo-splenic axis (Streilein and Niederkorn, 1981). The tolerizing effect can be transferred to naїve mice by serum (Griffith et al., 1995), and by circulating mononuclear cells (PBMC; Wilbanks and Streilein, 1992), from mice injected io with antigen some days previously. Some of these PBMC express the F4/80 antigen (Wilbanks and Streilein, 1992).

Trafficking studies using tagged molecules in mice injected io, or after application to the abraded cornea, indicate that soluble antigen rapidly reaches the eye-DLN within 30 min (Camelo et al., 2004, 2006) and then circulates widely to other lymph nodes (LNs; including the mesenteric) and to the spleen within several hours. Cell-associated antigen, after injection into the anterior chamber, can be detected in the DLN at 6 h, and in the spleen after 16–24 h indicating that cellular traffic from the eye to these sites is possible (Kuffová et al., 2008). However, data from experiments which involve intraocular injection require cautious interpretation since io penetration necessarily involves some backflow of antigen from the eye directly into the blood and lymphatic circulations (see below).

Irrespective of how the antigen reaches the spleen, i.e., as soluble, or later as cell-associated antigen, most groups agree that DTH-testable antigen-specific tolerance can be transferred to naїve mice by splenic CD8^+^ Tregs from mice injected io with antigen. The nature of the APC which promotes this tolerance is unclear, but mice deficient in cells expressing the macrophage surface marker F4/80, fail to generate tolerance after io injection (Lin et al., 2005). Intuitively, however, it is unlikely that cells from the eye directly mediate this effect since the number of ocular F4/80^+^ cells is limited; furthermore, previous studies failed to demonstrate APC migration from the iris after io antigen administration (Dullforce et al., 2004). An alternative possibility is that ocular fluids, perhaps containing material shed as microparticles or exosomes, or more conventionally as soluble proteins, from incoming inflammatory cells (including T cells undergoing Fas/FasL-mediated apoptosis and expressing TRAIL or shedding sTRAIL (Green and Ferguson, 2001; Griffith et al., 2011)), enter the blood circulation and arrive at the spleen where further amplification of the NKT cell/F4/80 spleen cell-mediated process of T cell apoptosis occurs (Faunce and Stein-Streilein, 2002). Since tolerance *via* this route is transferable by serum and has been shown to involve TCR components (Griffith et al., 1995), a possible explanation for T cell antigen specificity *via* cell surface particle shedding in this model arises.

The main flaw in these studies, going back to Medawar, is that the technical procedure of inoculating antigens into the eye causes breakdown of the blood–ocular barrier, however transiently, with leakage of antigen into the periocular blood and lymphatics, sufficient to permit rapid tracking of antigen to the spleen either directly *via* the blood or after a first pass through the DLN and thence to the spleen *via* the LN conduit system, high endothelial venules (HEV) and efferent lymphatics (Plskova et al., 2002; Kuffová et al., 2008). Tolerance induced by io injection is therefore not, in substance, different from tolerance induced by iv injection (antigen tracks directly to spleen), or by sc or ip injection (antigen tracks *via* DLN to the spleen). Models which suggest that TGFβ-treated F4/80^+^ peritoneal macrophages preferentially mirror ocular IP overstate the case (Hara et al., 1992; Niederkorn, 2009), and are probably not mechanistically different from models of myeloid suppressor cell activity (Gabrilovich and Nagaraj, 2009). Indeed, a recent study confirmed the role of splenic red pulp F4/80^hi^Mac-1^lo^ macrophages as immunosuppressive cells and showed that they act by inducing CD4^+^CD25^+^ Tregs in a CSF-1-dependent manner (Kurotaki et al., 2011). Gregerson et al. (2009) in an attempt to eliminate this technical flaw, showed that CD4^+^CD25^+^ Treg-mediated tolerance to endogenous retinal antigen, tested *via* retinal antigen-expressing viral infection, was reduced when the source of retinal antigen had been removed by enucleation of the eyes. However, the trauma of enucleation is likely to induce a systemic concomitant “Danger” signal which is difficult to control for (Miller et al., 2011; van de Veerdonk et al., 2011). In contrast, intact peripheral T cell anergy as well as CD4^+^CD25^+^ Treg generation (Lambe et al., 2007) seemed necessary to avoid spontaneous uveitis in a transgenic neoantigen model despite considerable central deletion (Lambe et al., 2007).

The time differential for soluble antigen (minutes) vs cell-associated antigen (hours) trafficking from the eye or indeed from any LN drainage site is important in the context of induction of tolerance vs immunity. LN resident APC, which will preferentially capture fast-tracking soluble antigen as it percolates along the conduits, present low levels of specific antigenic peptide–MHC Class II (p-MHCII) complexes, while migratory DCs entering the DLN several hours later present high levels of p-MHCII (Itano et al., 2003; Sixt et al., 2005). Both resident and migratory DCs have the capacity to induce T cell proliferation, but only migratory DC, through prolonged T cell/DC interactions, induce T cells which mediate DTH (for review, see Catron et al., 2004). Thus the earlier arrival of soluble antigen to the secondary lymphoid tissues will promote tolerance rather than DTH-style T cell responses. This underappreciated concept may explain many aspects of ocular IP and the essential nature of the time-dependent oculo-splenic axis since it may be indirectly accessed via rapid transit of “tolerizing” antigenic signals by a first pass through eye-DLN as well as directly through the bloodstream.

### SUBVERTING PRIVILEGE-PROMOTING CELLS

Despite the above caveats to what IP actually is and how it functions, there is little doubt that the intraocular microenvironment is immunomodulatory. Some of this is attributable to ocular-specific cells such as the iris–ciliary body epithelium and the RPE as shown in many *in vitro* studies, through mediators such as NO, PGE2, and retinoids (Forrester et al., 2008b; see Immunological Properties of the Eye). However, the microenvironment is altered by mediators introduced, e.g., *via* a disrupted BRB. Cytokines such as IFNγ, generated systemically during viral infections, can activate RPE cells to up-regulate immunosuppressive activity *via* PDL-1 (Ke et al., 2010) or to produce pro-inflammatory chemokines and cytokines locally, as well as induce MHC Class II expression on normally negative RPE (Liversidge et al., 1988, 1994; Mesri et al., 1994) and endothelial cells. Secretion of IL-6 by RPE cells in an environment rich in TGFβ, may be sufficient to convert CD4^+^ Tregs to Th17 cells and completely alter the immunosuppressive microenvironment to a pro-inflammatory one (Crane et al., 1999).

## BREACHES OF PRIVILEGE

Immune privilege comes at a cost – if the privileged status of the eye is compromised, the ensuing disease can be devastating (Caspi, 2006; Forrester et al., 2008b). Several ocular conditions occur in which IP fails.

### NON-INFECTIOUS INTRAOCULAR INFLAMMATION (UVEITIS)

Uveitis is a common disease (Darrell et al., 1962; Gritz and Wong, 2004; Williams et al., 2007) and comes in several varieties (**Box 1**). Direct infectious uveitis is less common while non-infectious uveitis is presumed to be (auto)immune in character. Despite their low expression on ocular cells, certain forms of uveitis have strong links with MHC Class I antigens (HLA B27: acute anterior uveitis; HLA B51: Behcet’s disease; HLA A29: birdshot retinochoroiditis; Levinson, 2007). Acute anterior uveitis may be self-limiting, which has been attributed to Fas/FasL-induced T cell apoptosis, demonstrating IP in action in human disease (Dick et al., 1999).

Non-infectious uveitis is CD4^+^ T cell-mediated (Th1 and Th17) in humans and mice (Amadi-Obi et al., 2007; Caspi, 2010). Ocular tissues contain many potential autoantigenic targets, especially retina, apparently sequestered from the immune system (Gregerson et al., 2009). Consequently, escape of antigen during damage (trauma, infection, inherited degenerations) is one route to activate rare autoreactive T cells in the periphery (Caspi, 2010). However, most uveitis occurs in virgin tissue, raising the question: how do activated T cells cross the BRB? Systemic signals, from chemokines and other molecules, appear to “pre-pare the way” across the endothelium (Xu et al., 2004, 2008; Crane et al., 2006).

“Systemic signals” generated during infections, involving mechanisms such as molecular mimicry and bystander activation (Benoist and Mathis, 2001; Ji et al., 2010), do not disguise the fact that, once activated, antigen-specific T cells enter the retina and cornea with as much ease as into any tissue, accumulate *in situ*, and attract pro-inflammatory macrophages which cause tissue damage (Forrester et al., 1998). In EAU, inflammation declines inversely with an increase in CD4^+^CD25^+^FoxP3 Tregs, which accumulate and promote resolution. However, EAU in the C57/BL6 mouse does not completely resolve but persists with a macrophage-mediated choroidoretinal angiogenic response (Chen et al., 2012) much as occurs in some uncommon human uveitides (PIC, see **Box 1**; Atan et al., 2011).

Spontaneous models of EAU more closely represent non-infectious human disease, which occurs without a recognizable trigger. Limited T cell anergy as well as possible antigen escape from sequestration (immunological ignorance) have been suggested to underpin breakdown of tolerance in this model (Lambe et al., 2007; Gregerson et al., 2009). Interestingly, early cells entering the tissues in this model are IL-17-secreting γδ-like T cells (Makinen, 2006), which have been implicated in the early pathogenesis of conventional IRBP-induced EAU (Nian et al., 2011). However, γδ T cells may have a regulatory role (Girardi and Hayday, 2005; Pennington et al., 2005; Nian et al., 2010) as well as a pathogenic role (Nian et al., 2011) in autoimmune uveoretinitis.

Immune privilege appears not to afford much protection against the damaging effects of uveitis possibly because IP works at the level of tissue homeostasis, mainly keeping healthy tissue free of random migrants which might provoke inflammation. However, when faced with a serious challenge, IP dismally fails to prevent severe destruction: in uncontrolled sight-threatening uveitis, both infection and the immune response to infection, can cause permanent structural damage. Therapies such as anti-TNFα disable the destructive effects of inflammation while permitting harmless monocytes to traverse the tissues without causing damage (reviewed in Khera et al., 2010).

### TRANSPLANTATION

Several types of ocular allografts are performed in humans, including corneal, limbal (stem cells), scleral, and retinal grafts. However, IP does not protect against allograft rejection: even artificial corneas constructed from pig collagen, are rejected *via* antibody-mediated mechanisms (Liu et al., 2007).

#### Corneal Grafts

Despite a long-established reputation for high rates of acceptance, long-term corneal graft survival rates actually lag behind vascularized solid-organ grafts (Williams and Coster, 1997). Corneal grafts in humans are normally performed without tissue matching, and graft acceptance was historically attributed to the absence of passenger leukocytes. However, as indicated above, corneal tissue contains both resident MHC Class II^+^ macrophages and peripheral lymphatics (Brissette-Storkus et al., 2002; Sosnova et al., 2005; Xu et al., 2007a; Ecoiffier et al., 2010). Despite the presence of MHC Class I and II leukocytes in the donor cornea, corneal graft rejection occurs at a slower tempo than comparably unmatched solid-organ allografts indicating a degree of “privilege”; in part this is due to the fact that corneal graft rejection is mediated *via* indirect allorecognition, and direct alloresponses do not contribute to this process (Boisgerault et al., 2009). Corneal graft rejection thus resembles chronic indirect allorecognition of vascularized grafts which is also of a considerably slower tempo, is mediated by CD4 T cells, and is greatly accelerated when innate immune activation is at high levels, e.g., in cases of infectious or atopic ocular surface disease, in both of which vascularization is prominent (Niederkorn, 2010). CD4 Th1 cells are the main pathogenic T cells which induce corneal allograft rejection, although recent evidence has implicated Th2 cells; intriguingly there is a suggestion that IL-17A, whether derived from Th17 cells or from other sources, is necessary for allograft survival (reviewed in Cunnusamy et al., 2010). The evidence for IP preventing corneal graft rejection is therefore not strong, its main effect probably being to blunt direct allorecognition. Perhaps the most intriguing possibility is that the absence of a strong CD8^+^ T cell cytolytic alloresponse in orthotopic corneal graft rejection (Boisgerault et al., 2009) may not simply be attributable to low levels of donor MHC antigens, but that donor leukocytes migrating to the host DLN, arrive there as “privileged” cells from a healthy thrombospondin (TSP)-, RA-, and TGFβ-rich donor immunoregulatory microenvironment (Saban et al., 2010) and are more liable to promote tolerance rather than immunity. The balance will be decided by the level of inflammation and innate immunity at the site of the graft and thus the degree of surgical manipulation and associated trauma itself will influence the outcome. In addition, corneal expression of immunoregulatory molecules such as FasL and TRAIL help to promote this IP/TCT (Ferguson and Griffith, 2007). One major contributor to the immunoregulatory microenvironment appears to be DAF (CD55) expressed by both donor and host corneal cells (Esposito et al., 2010). However, this is insufficient to fully prevent the indirect alloresponse, which continues to fuel the rejection process.

#### Retinal Transplants

Several attempts have been made to transplant retinal tissue (Anosova et al., 2001) or cells (photoreceptor cells, West et al., 2010; Gust and Reh, 2011; RPE cells, Tezel et al., 2007) to repair damaged or degenerating retina (West et al., 2009), but only occasionally has survival and, importantly neural integration, been reported (MacLaren et al., 2006). The erroneous notion that ocular IP will promote acceptance of such grafts has been exposed by the need for immunosuppression to assist graft acceptance (West et al., 2010). Very recently, photoreceptor transplantation, neural integration, and even evidence of visual function in mice have been demonstrated, indicating the feasibility of such an approach although information on the duration of survival of the photoreceptors was not detailed (Pearson et al., 2012). In a similar study, photoreceptor survival was found to extend for 4 weeks after which there was progressive apoptosis of the grafted cells in the absence of significant inflammation. Survival could be extended by prior transfection of the pre-cursor photoreceptor cells with XIAP, an anti-apoptotic gene (Yao et al., 2011).

Intraocular stem cell transplants have been used to encourage retinal cell differentiation in the appropriate microenvironment, but also to capitalize on the immunosuppressive properties of certain cells, such as mesenchymal stem cells (MSCs). It is too early to decide whether these approaches are fanciful, but systemic immunosuppression is required to delay rejection of such grafts even for a short time, and accepted grafts do not appear to integrate with retinal neuronal circuits (Hill et al., 2009; West et al., 2010).

### OCULAR INFECTIONS: LESSONS LEARNT FROM AIDS

Infectious uveitis is unusual in the absence of systemic immunodeficiency. Many infectious organisms such as *Toxoplasma gondii* and herpes simplex virus, reside latently in privileged sites such as the eye and brain (Kinchington et al., 2012); however, the relative sanctuary provided by these sites is still under some degree of systemic immune control since it is not until the CD4 T cell is disabled, as in untreated AIDS patients, that these organisms can “reactivate” and replicate, thereby causing severe damage (Scholz et al., 2003). Organisms include toxoplasma, mycobacteria, pneumocystis, candida, and other fungi, and several herpes viruses such as CMV, HSV, and HTLV-1. Such organisms survive and persist but “hide” from the immune system by hijacking privilege (Jones et al., 2006; Lepisto et al., 2007). Interestingly, the new phenomenon of “immune recovery uveitis” in HAART-treated AIDS patients indicates that once the eye’s guard is lowered, it is susceptible to immune-mediated damage (Jabs, 2011), thus revealing that the privileged status of the eye is an active process requiring continued maintenance. Immune-mediated damage in these circumstances can also be the result of reactivated infection, thus complicating the issue further (Moorthy et al., 2012).

These developments in the context of AIDS have relevance for non-AIDS-associated infectious uveitis in immunocompetent patients. For instance much of the sight-threatening effects of infectious disease (including the full panoply of viral, (myco)bacterial, parasitic, and fungal infections such as trachoma, onchocerca, toxoplasmosis, herpes stromal keratitis in which there can be extensive tissue damage), is caused by a robust or even exaggerated immune response. Thus although the host survives, blindness may be the cost. The corollary is also true, as evidenced by the failure of IP in an aging immune system to protect against the re-awakening of latent infectious disease, much of which was contracted in the neonatal and early childhood years. Interestingly, those rare fatal cases of CNS HSV infection in children appear to be linked to a mutation in TLR3, providing an example from Nature in which defective innate immunity tips the balance in this precarious struggle between immunity and infection (Zhang et al., 2007; Guo et al., 2011).

## PRIVILEGE IN THE BALANCE – PARA-INFLAMMATION

It is clear from the above that ocular IP is a finely judged act which can readily tip. This applies to many physiological processes and Medzhitov (2008) described it neatly as para-inflammation when considering the body’s response to predictable perturbations such as aging.

### DEGENERATIVE RETINAL DISEASE AND THE OCULAR IMMUNE RESPONSE

**FIGURE 4:**
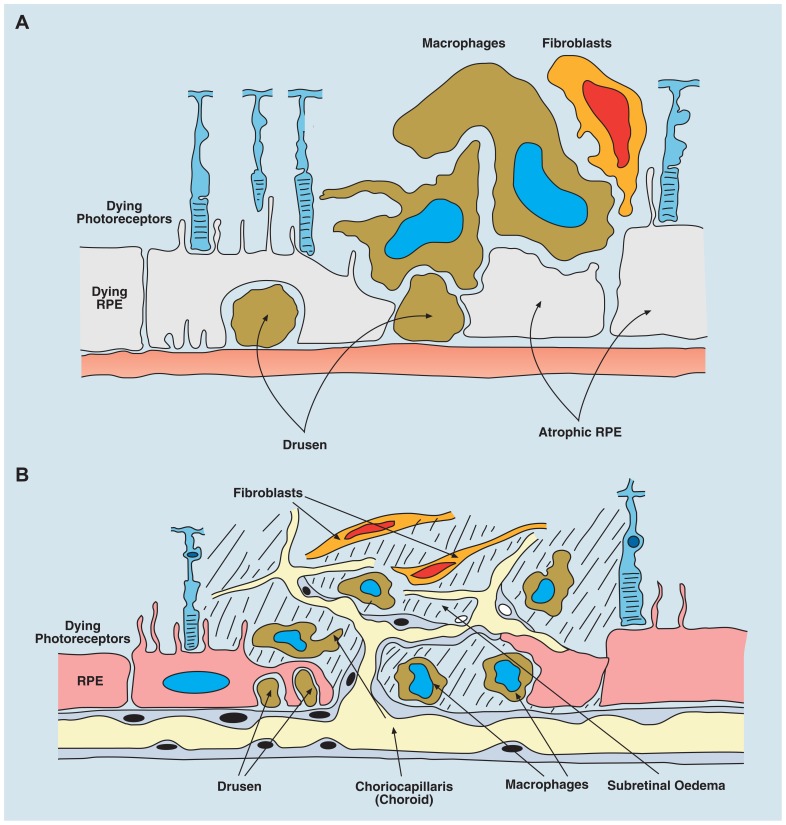
**Age-related macular degeneration (AMD) occurs in two forms: dry, atrophic damage to the retinal photoreceptor and RPE cells with chronic progressive loss of visual acuity; and wet AMD in which new vessels originating from the choroidal layer, penetrate the basement membrane of the RPE cells and leak fluid or blood into the subretinal space with rapid loss of vision**. **(A)** Diagram of dry AMD showing drusen under the RPE layer, and extensive atrophic damage to RPE cells. **(B)** Diagram of wet AMD showing in-growth of new blood vessels into the subretinal space, causing extensive destruction of RPE and photoreceptor layers.

Removal of dead and dying cells is homeostatic tissue husbandry. Ferguson has championed this process to explain systemic tolerance induced after io injection of antigen, and regards it as a general phenomenon (Ferguson and Griffith, 2007; Griffith et al., 2011). Dead-cell scavenging occurs throughout life, and depends on innate immune mechanisms involving resident macrophage and parenchymal cell activity. AMD is considered a disease in which para-inflammatory mechanisms fail either for genetic (defects in complement genes) or environmental (smoking, diet, metabolic disturbance) or even chronic infective (chlamydia, HSV) reasons (reviewed in Xu et al., 2009). AMD is characterized by the accumulation of waste products (drusen) presumably from retinal photoreceptor cells, both inside (increased lipofuscin accumulation) and below the RPE cell (**Figure 4A**), in part due to impaired complement Factor H binding to sulfated glycosaminoglycans (Clark et al., 2010). Resident F4/80^+^ macrophages and DCs in the sub-RPE/choroid (**Figure 1**) assist in clearing drusen (Forrester et al., 2010); however, when drusen accumulate, there is deposition of complement components and other acute phase proteins, leading to a low grade pro-inflammatory macrophage response and eventual subretinal neovascularization (Skeie and Mullins, 2009; **Figure 4B**). RPE cells normally produce anti-angiogenic factors (pigment epithelial cell-derived factor, PEDF and TSP), but can switch to produce pro-inflammatory factors such as VEGF, which promote wet AMD (Ablonczy et al., 2009). Prior to this (aberrant) reparative angiogenic response however, chronic age-related degenerative changes in the RPE occur and atrophic (Guo et al., 2011) AMD ensues, possibly as a consequence of a defect in intracellular microRNA regulation via DICER1 (Kaneko et al., 2011). In fact recent evidence suggests that whether AMD develops as the dry or wet form reflects how the “privileged” retina responds to specific mediators. In dry AMD, DICER appears to act via induction of the inflammasome through NLRP3 and secretion of IL-18, leading to RPE cell death, the hallmark of geographic atrophy or dry AMD (Tarallo et al., 2012). In contrast, cells which phagocytose drusen, the characteristic waste product produced by the RPE cell, also secrete increased amounts of IL-18 again through NLRP-mediated activation of the inflammasome, but under these circumstances IL-18 may protect against retinal angiogenic responses, the hallmark of wet AMD and the obverse of geographic atrophy (Doyle et al., 2012). Thus it appears, in this specific instance, that IL-18, which is constitutively produced by the RPE cell (Jiang et al., 2001), acts as a regulator of RPE function and determines whether the RPE cell succumbs to aging stress (para-inflammation) and dies (geographic atrophy) while attempting to prevent an angiogenic response to an increasing age-related pro-inflammatory microenvironment.

The angiogenic response in AMD is similar to other intraocular angiogenic responses such as the neovascular membranes which occur in low grade chronic uveitis, and may be promoted by arginase^+^ macrophages, as opposed to immunoregulatory (F4/80^+^) macrophages associated with IP. Thus IP can be breached not only by severe overwhelming inflammatory disease, but by chronic, low grade angiogenesis-associated inflammation as in chronic uveitis and AMD. Moreover, pathological subretinal neovascularization can be reversed by utilizing IP-promoting properties of FasL (Roychoudhury et al., 2010) and it may also be true that IL-18 produced by the RPE is a further IP-associated mediator of a constitutive anti-angiogenic response (Jiang et al., 2001; Doyle et al., 2012).

Thus there is a spectrum of responses from homeostasis and health (intact IP) through aging and chronic disease (disabled IP) to fulminant (infectious) retinal necrosis (absent IP). Much of this activity is considered “auto-inflammatory” involving activation of innate immune receptors on various subsets of macrophages, but an adaptive immune response in AMD, involving lipid or proteolipid antigen and CD1a may also contribute to AMD-like disease (Hollyfield et al., 2008). This has implications for potential stem cell therapies currently being mooted for treatment of inherited retinal disease, and even AMD, since the assumption that IP protects intraocular stem cell inocula from scrutiny by the immune system is clearly not true. As discussed below for glaucoma, the ultimate test of an immunological basis for AMD will depend on whether an immunologically based therapy will modify the disease.

### GLAUCOMA AND RETINAL NERVE DAMAGE

Chronic open angle glaucoma is a disease where cellular waste accumulates in the trabecular meshwork (aqueous drainage pathway, **Figure 1**) and may initiate an inflammatory response (Wax, 2011). While the evidence for this is scanty, the outcome of glaucoma is neuroretinal damage which, when mediated by the amino acid glutamate, is accompanied by a prominent inflammatory response. Remarkably, however, the retinal response to glutamate damage appears to be one of enhanced neuroprotection mediated by recruitment of myeloid-derived suppressor cells (MDSCs) (London et al., 2011). Indeed glutamate has been shown to have IP properties at least in the brain (Fallarino et al., 2010; Hansen and Caspi, 2010). There is a need for good experimental models of glaucoma which truly reflect the human disease. A popular current model involves thermal or hypertonic occlusion of episcleral veins, thus preventing aqueous drainage. The associated raised intraocular pressure leads to ganglion cell damage in which activated CD200R^+^ retinal microglia are implicated (Taylor et al., 2011).

In addition to the need for a suitable model of experimental glaucoma which would reflect the human disease, the complex role of IP-mediating factors is revealed. In a spontaneous model of glaucoma, the absence of sFasL, which is a cleavage product of membrane bound FasL, was shown to be associated with increased retinal ganglion cell death while administration of sFasL to the same mice, protected them from damage (Gregory et al., 2011).

The evidence for involvement of the immune system in glaucoma is tenuous but suggestive. Numerous studies in humans both of systemic factors and of ocular tissue obtained from patients, show several features such as potential serum indicators of disease as well as signs of innate immune activation of glial cells with increased expression of MHC class II and other activation markers (reviewed in Tezel, 2011). However, whether this is genuine evidence for an adaptive immune response in glaucoma, or simply an up-regulated innate immune response to damage as occurs in para-inflammation, is a moot point. As for AMD, the test will come when an immunologically based therapy shows an effective protection against the progressive vision-destroying consequences of glaucoma.

### DIABETIC RETINOPATHY

Diabetic retinopathy (DR) is the fourth commonest cause of world-wide blindness, potentially increasing as the epidemic of diabetes expands (Suttorp-Schulten and Rothova, 1996). DR is a microvascular endotheliopathy, affecting small capillaries and post-capillary venules leading to occlusion and expanding areas of retinal ischemia (Grant et al., 2004; Busik et al., 2009; Bhatwadekar et al., 2010). Like aging, diabetes leads to increasing levels of intravascular leukocyte activation and adhesion, contributing to capillary occlusion (MacKinnon et al., 2004). This probably involves platelet–monocyte interactions (Ogata et al., 2006) since there is evidence that CCR5^+^CD11b^+^ monocytes are the culprit leukocytes (Serra et al., 2012). Trapped monocytes, as well as activated retinal microglial and other retinal cells are sources of VEGF driven by HIF1α (Wang et al., 2010; You et al., 2010), and activate retinal angiogenesis, producing the vision-destroying late disease, proliferative DR (PDR). Thus, drugs such as statins act not only on metabolic lipid pathways (Klein, 2010) but also as inhibitors of leukocyte adhesion to ameliorate or delay disease (Greenwood and Mason, 2007) which has been confirmed in a model of DR (Serra et al., 2012). The ocular pathology is not specific but reflects a general process, also affecting kidneys and peripheral nerves; indeed direct effects on bone marrow-derived hemopoietic stem cell precursors accounts for the poor overall wound healing response in diabetes (Busik et al., 2009). PDR in fact represents a last-ditch but misguided attempt by the retina to repair itself.

An interesting convergence of dysregulated metabolism and activation of immune cells has emerged through the discovery of the succinate receptor (SUCNR1; reviewed in Ariza et al., 2012) which is likely to impact on the pathogenesis of diabetes and its complications. Succinate is a normal metabolite involved in the citric acid cycle and, in times of stress, accumulates in the mitochondria and finds its way to the extracellular space through a series of transporters and porins in the various cell membranes. Extracellular succinate levels above a certain concentration activate SUCNR1 on immature DCs and macrophages and lead to induction of the inflammasome via accumulation of HIF1α even in normoxic conditions (Wen et al., 2012).

The SUCNR1 receptor is expressed in the retina, specifically in the retinal ganglion cells and in the RPE cells and has been identified as having a major role in angiogenesis in the developing retina and also possibly in the retinal ischemia associated with DR (Sapieha et al., 2008). The role of immune cells, particularly inflammatory macrophages in retinal angiogenesis is well-known (Chen et al., 2012) and the possibility that succinate may facilitate, if not drive, theses responses in pathological conditions such as diabetes and perhaps in inflammation generally is an intriguing one.

## WHEN PRIVILEGE IS DANGEROUS

Immune privilege allows the eye “to keep a clean house,” but may compromise survival of the host, for instance through unchecked growth of tumors or through uncontrolled viral replication in the CNS.

### INTRAOCULAR MELANOMA

The commonest primary tumor of the eye is melanoma but tumor growth is slower than in other tissues and the risk of tumor spread is less; ocular melanoma occurs in older patients and late metastases are commoner than in cutaneous melanoma (Kujala et al., 2003; Rigel et al., 2010). The mode of spread in part determines metastatic risk, ocular melanoma spread being predominantly hematogenous, while cutaneous melanoma risk is determined by local invasive depth (Rigel et al., 2010). Unchecked growth of non-ocular tumors correlates with immune suppression, also described as a form of IP (Kandalaft et al., 2010), and ascribed in ocular tumors to tumor-infiltrating Tregs (Mougiakakos et al., 2010). IP-related mechanisms may not only allow active growth of allogeneic tumors grafted intraocularly, but eventually facilitates tumor metastasis, leading to death (Niederkorn, 1997). However, experimental models of intraocular tumors do not precisely mirror the condition in humans, in particular by the important fact that the experiment necessarily requires breakdown of the ocular barrier which is the critical factor determining metastases in humans. Despite or perhaps in the light of this caveat, it is somewhat surprising that intraocular melanomas in humans fail to grow as rapidly as skin melanomas. The behavior of intraocular tumors is related to the density of intratumoral macrophages (reviewed in Jager et al., 2011) and depends on their type and angiogenic properties: ocular melanoma pathogenicity is predicated on its vascularity both clinically and experimentally (Foss et al., 1996; McKenna and Kapp, 2006) while removal of M2-type macrophages almost completely prevents tumor growth (Ly et al., 2010). It is interesting that IP of non-ocular tumors is also attributed to vascularity and to VEGF expression (Kandalaft et al., 2010). In addition, intratumoral macrophages may belong to the myeloid-derived suppressor cells (MDSCs) variety (Natarajan and Thomson, 2010; Bronkhorst et al., 2011; Chioda et al., 2011) and directly suppress anti-tumor T cell responses. These tumor-IP related cells have been identified in the spleen as GM-CSF-dependent, CD11b^+^Gr1^lo/int^ myeloid cells (Dolcetti et al., 2010; Ugel et al., 2012) and it is interesting to speculate whether they are equivalent to the spleen F4/80^+^ macrophages identified as potential mediators of ocular IP (see above). In contrast, in other ocular tumors, at least experimentally, pro-inflammatory macrophages promote tumor regression (McKenna and Chen, 2010) and are recruited from a population of CD11b^+^CD15^+^ granulocytic cells in the circulation (McKenna et al., 2009). However, these cells are associated with Treg induction and their precise role is unclear. Interestingly, Tregs in some non-ocular tumors may have a beneficial effect by regulating MDSCs, revealing the complexity of cellular interactions within the tumor microenvironment (Zhang et al., 2010). The “privileged” tumor microenvironment may actually co-operate with ocular IP. However, while a synergistic “immunoregulatory” double-dose of IP may be operative, tumor IP and ocular IP may alternatively cancel each other out, allowing either an uncontrolled profound pro-inflammatory immune response, with spontaneous regression of the tumor and severe collateral intraocular inflammation, or a super-suppressed, profoundly anergic anti-tumor response with metastases and rapid extraocular spread (i.e., a failure of response). The former is less likely than the latter but is well-documented clinically and experimentally (Knisely and Niederkorn, 1990; Shields et al., 1999). In addition, several mechanisms exist for tumor rejection or evasion by the immune system. In one series of experiments, rejection of tumors in the mouse anterior chamber of the eye was dependent on CD4^+^ T cells and TRAIL, the same molecule which is considered to underpin ocular IP-related systemic immune tolerance (see above; Wang et al., 2003). A further regulatory element in IP-mediated growth of intraocular tumors is *via* the sympathetic nervous system which in one experiment was seen to be closely linked to intraocular production of TGFβ (Vega et al., 2009).

Not all intraocular tumors behave similarly even when derived from similar cells: many follow the pattern of growth underpinned by privileged immunity while others are rejected. Recently it has been shown that a specific tumor in mice, Ad5E1 can follow two patterns of rejection one of which involves severe rejection and tumor necrosis mediated by IFNγ with severe destructive bystander damage to the eye which is dependent on TNFα, while the second pattern also leads to tumor rejection but there is minimal ocular damage. In the latter case, tumor killing was mediated by an arginase^+^ population of macrophages (Coursey et al., 2012). Thus direct killing via CTLs or by different subsets of macrophages appears to determine the outcome both for the tumor and for the eye (for review, see McKenna and Chen, 2010). Similar cellular diversity has been found in intraocular melanomas in humans (Bronkhorst et al., 2012).

It can be seen from this discussion that the immune response to ocular tumors hangs in the balance, as does the survival of the host. This precarious condition is dictated by the strength of the ocular IP effect vs the desirable but impaired, anti-tumor response. For tumors arising in the eye such as melanoma, the combined effect of these “privileged” responses is beneficial both to the eye and the host provided the tumor is contained within the eye, but once it breaks free the desired systemic immune response is inadequate to control the tumor with fatal consequences in many cases.

### PRIMARY INTRAOCULAR LYMPHOMA

The eye contains few B and T cells outside the vasculature, occasional cells passing through the fenestrated walls of the uveal vessels (**Figure 1**). Primary intraocular lymphoma is grouped with CNS lymphoma, a rare extra-nodal variant of non-Hodgkin’s lymphoma (Algazi et al., 2009) arising from post-germinal center B cells (Coupland et al., 2009) and occurs by seeding of privileged sites by hemopoietic progenitor cells after variable lineage differentiation. The precise location of the lymphoma predicts its behavior with retinal lymphomas being aggressive and choroidal lymphomas being more “indolent,” while iris and ciliary body lymphomas are very rare (Coupland and Damato, 2008; Mashayekhi et al., 2012). This behavior may reflect the relative IP status of the tissue, retina being likely to posses greater privilege than choroid, perhaps determined by specific subsets of resident myeloid cells (see Cells and Tissues).

Primary intraocular tumors may represent one aspect of “an experiment of Nature.” The paradoxically beneficial effect of Treg cells (see above) in some tumors of the head and neck is clearly demonstrated by the development of tertiary lymphoid structures (TLS) surrounding the tumor. TLS express intense immunoregulatory activity directed toward reducing chronic inflammation, a recognized poor prognostic sign, and have been likened to sites of induced IP. Constitutive IP in the eye may behave like a “TLS” to protect the host in cases of intraocular lymphomas but is a dangerous strategy (Fridman et al., 2010).

However, due to the rarity of primary intraocular lymphoma, their biology is poorly understood and animal models have not been very informative so far, thus the call for multicenter studies to investigate these tumors is timely (Chan et al., 2011).

### CANCER-ASSOCIATED RETINOPATHY AND THE PARA-NEOPLASTIC SYNDROME

Another experiment of Nature is revealed by cancer-associated retinopathy (CAR). Retinal antigens aberrantly expressed in extra-ocular tumors induce serum antibodies and T cell responses, invoking a para-neoplastic syndrome of progressive retinal degeneration and eventual blindness (Bazhin et al., 2007). Early studies revealed that antigens such as the photoreceptor visual cycle regulatory protein, recoverin, were responsible and many further retinal antigens including the potent autoantigen, IRBP (see above) have been implicated (Bianciotto et al., 2010). These observations spawned more intense investigations into (auto)immune-mediated causes of common retinal degenerations such as AMD, and prompted much of the immunological studies which came in the wake of knowledge concerning the role of innate immune genes such as complement. Interestingly, an alternative theory involving secretion of VEGF by the tumor and expression of VEGFR1 by retinal neural and vascular cells has been proposed to explain CAR syndrome which involves specifically loss of pericytes and increased retinal vascular leakage (Cao and Cao, 2010).

Cancer-associated retinopathy also provides insight into the nature of ocular IP and supports the notion of immunological ignorance (antigen sequestration) as one form of IP. However, the discussion above clearly shows that “all roads lead to Rome” and that ocular IP, just like immunological tolerance generally, is subject to many checkpoints. It is not intrinsically diff-erent from other forms of immune tolerance (Matzinger and Kamala, 2011).

## CONCLUSION

Immune regulation/tolerance induction occurs primarily *via* central and peripheral mechanisms. However, there is an evolving understanding that the target tissue and its microenvironment can also modify the immune response (Matzinger and Kamala, 2011) and how this develops depends on the nature and properties of the tissue. The eye provides an excellent paradigm for the concept of tissue regulation of immune responses and how the same “danger signals” might not be recognized in the eye as they are, for instance, in the lung or skin. Thus, the violent immune response to toxoplasma in the gut is much more subdued in the eye, giving the parasite time to sequester itself from the patrolling killer immune cells. However, the process is not failsafe and when it fails, it fails gloriously, with irreparable damage to ocular structures and loss of sight (e.g., HSV-induced acute retinal necrosis).

The ocular immune response encapsulates the full range of classical and non-classical immune responses and demonstrates many features which are reflected in other tissues, but eye tissues by modifying these responses reveal unexpected and novel features, which are relevant to immune responses generally. In addition, IP involves many recognized immunoregulatory processes, including induction of Tregs, and is inducible and transferable. This has therapeutic potential, particularly for devising ways to restore tolerance in ocular inflammatory disease, and for preventing rejection of cells and tissues, such as stem cells, currently being considered for treatment of world-wide blinding diseases such as AMD.

## Conflict of Interest Statement

The authors declare that the research was conducted in the absence of any commercial or financial relationships that could be construed as a potential conflict of interest.

## Acknowledgments

The authors thank Drs Mariapia Degli-Esposti and Matt Wikstrom for critical reading of the manuscript. Original research by the authors referenced in this article was supported by the Development Trust of the University of Aberdeen and Action Medical Research, UK. We also thank Mr. Gordon Stables for the design and preparation of the figures.
